# A Novel Platelet-Activating Factor Receptor Antagonist Inhibits Choroidal Neovascularization and Subretinal Fibrosis

**DOI:** 10.1371/journal.pone.0068173

**Published:** 2013-06-27

**Authors:** Han Zhang, Yang Yang, Atsunobu Takeda, Takeru Yoshimura, Yuji Oshima, Koh-Hei Sonoda, Tatsuro Ishibashi

**Affiliations:** 1 Department of Ophthalmology, Graduate School of Medical Sciences, Kyushu University, Fukuoka, Japan; 2 Department of Ophthalmology, the First Hospital of China Medical University, Shenyang, China; 3 Department of Ophthalmology, Yamaguchi University Graduate School of Medicine, Ube, Yamaguchi, Japan; Leibniz Institute for Age Research - Fritz Lipmann Institute (FLI), Germany

## Abstract

Choroidal neovascularization (CNV) is a critical pathogenesis in age-related macular degeneration (AMD), the most common cause of blindness in developed countries. To date, the precise molecular and cellular mechanisms underlying CNV have not been elucidated. Platelet-activating factor (PAF) has been previously implicated in angiogenesis; however, the roles of PAF and its receptor (PAF-R) in CNV have not been addressed. The present study reveals several important findings concerning the relationship of the PAF-R signaling with CNV. PAF-R was detected in a mouse model of laser-induced CNV and was upregulated during CNV development. Experimental CNV was suppressed by administering WEB2086, a novel PAF-R antagonist. WEB2086-dependent suppression of CNV occurred via the inhibition of macrophage infiltration and the expression of proangiogenic (vascular endothelial growth factor) and proinflammatory molecules (monocyte chemotactic protein-1 and IL-6) in the retinal pigment epithelium–choroid complex. Additionally, WEB2086-induced PAF-R blockage suppresses experimentally induced subretinal fibrosis, which resembles the fibrotic subretinal scarring observed in neovascular AMD. As optimal treatment modalities for neovascular AMD would target the multiple mechanisms of AMD-associated vision loss, including neovascularization, inflammation and fibrosis, our results suggest PAF-R as an attractive molecular target in the treatment of AMD.

## Introduction

Neovascular age-related macular degeneration (AMD) leads to severe deterioration of central vision in elderly individuals owing to the development of choroidal neovascularization (CNV) in the macular region [1]. Abnormal new blood vessels initially proliferate under Bruch's membrane and the retinal pigment epithelium (RPE) and then invade the subretinal space, leading to subretinal hemorrhages, exudative lesions, serous retinal detachment, and ultimately disciform scarring [2]. Local destruction of photoreceptors, RPE, and choroidal blood vessels leads to irreversible loss of macular function and vision.

CNV is regarded as a submacular wound healing process that requires a continually evolving interaction among cells, cytokines, and the extracellular matrix [2], [3]. Angiogenesis is an essential component of this process, and current clinical strategies for treating CNV are primarily aimed at inhibiting vascular endothelial growth factor (VEGF), the major promoter of angiogenesis [4], [5]. However, overall only 30%–40% of exudative AMD patients gain three lines in visual acuity, and approximately 1 in 6 patients experience progressive loss in visual acuity that leads to legal blindness despite standard treatment with potent VEGF inhibitors [6]–[8]. These results are not surprising because angiogenesis is only one component of the wound healing process and because CNV pathogenesis extends beyond the endothelium. Therefore, CNV may be amenable to additional therapeutic alternatives besides anti-angiogenesis. During the past decade, several studies have examined the immune mechanisms in AMD and have reached the consensus that inflammation is a key driver in the development of neovascular AMD [2], [3], [9]–[11]. AMD is regarded as the result of an ongoing low-grade chronic inflammatory process, much like Alzheimer's disease and other chronic diseases of aging. This inflammatory process includes macrophage infiltration and the regulation of cytokine networks, which mediate CNV development [9].

Platelet-activating factor (PAF, 1-O-alkyl-2-acetyl-sn-glycero-3-phosphocholine), the first bioactive lipid ever identified, is a potent proinflammatory mediator that is involved in cellular activation, intracellular signaling, apoptosis, and diverse inflammatory reactions [12]–[15]. Its biological actions are mediated through the activation of a G protein-coupled PAF receptor (PAF-R) [16]. Several studies have suggested the involvement of PAF in angiogenesis. PAF directly stimulates the *in vitro* migration of endothelial cells, enhances vascular permeability, and promotes *in vivo* angiogenesis [17]–[21]. The results of animal studies suggest that PAF may contribute to the angiogenic activity of certain cytokines by stimulating the production of VEGF, tumor necrosis factor-alpha, and hepatocyte growth factor [19], [22], [23]. A recent study shows that PAF-R is present in RPE cells and choroidal endothelial cells, and PAF upregulates VEGF in RPE cells [24]. Because these cell types are important for CNV development, these findings suggest that PAF may be involved in the pathogenesis of neovascular AMD. However, *in vivo* evidence supporting the role of PAF and PAF-R in CNV has not been reported.

In the present study, we demonstrate that local expression of PAF-R in the subretinal space is upregulated during CNV development. Administration of the PAF-R antagonist potently attenuated CNV lesion size by suppressing macrophage infiltration and the expression of multiple CNV-related molecules in the injured eye. We further report that PAF-R blockage inhibits experimental subretinal fibrosis. Thus, PAF-R blockage may provide a novel, effective treatment for neovascular AMD.

## Materials and Methods

### Animals

Female 7- to 10-week-old C57BL/6 mice were purchased from Japan SLC (Shizuoka, Japan) and used in all experiments. All animal experiments were approved by the Committee on the Ethics of Animal Experiments, Graduate School of Medical Sciences, Kyushu University, Japan. Animals were treated according to the ARVO Statement for the Use of Animals in Ophthalmic and Vision Research.

### Induction and Evaluation of CNV

CNV was induced by photocoagulation as described previously, with some modifications [25]. In brief, laser photocoagulation was applied around the optic disc using a 532-nm diode laser (200 mW, 0.1-s duration, 75-µm diameter; Iridex, Mountain View, CA) to burn the posterior pole of the retina (4 spots/eye). Only lesions in which a subretinal bubble developed were used in subsequent experiments. Ten days after photocoagulation, the mice were anesthetized and perfused with 50 mg/ml of fluorescein-labeled dextran (2×10^6^ average molecular weight; Sigma, St. Louis, MO). Following enucleation and fixation in 4% paraformaldehyde, corneas and lenses were removed, and each entire retina was dissected from the eye cup. After each eye cup was flat-mounted, the total area of hyperfluorescence associated with each burn was measured using ImageJ software (National Institutes of Health, Bethesda, MD).

### Treatment with PAF-R Antagonist

Animals were treated with the PAF-R antagonist WEB2086 (Santa Cruz Biotechnology, Santa Cruz, CA) or phosphate-buffered saline (PBS, used as vehicle) 1 h before photocoagulation, and treatments were continued daily until the end of the study. WEB2086 was administered intraperitoneally to mice at 5 mg/kg body weight.

In some experiments, mice were injected intravitreally with 1 µg WEB2086 in 0.5 µl of PBS immediately after laser photocoagulation using 32-gauge needles (Hamilton, Reno, NV) under an operating microscope. Injections were repeated every other day for the duration of the study.

### Immunohistochemistry and Immunofluorescence

For PAF-R staining, cryostat sections (6-µm thick) from naïve and laser-treated eyes (5 days after photocoagulation) were incubated with polyclonal rabbit anti-PAF-R antibody (1∶400, Santa Cruz Biotechnology) and bovine anti-rabbit IgG-HRP (Santa Cruz Biotechnology), followed by the chromogen AEC (Vector Laboratories, Burlingame, CA). To bleach the pigment in RPE and choroid, the sections were incubated in 3% H_2_O_2_ for 18 h at room temperature and then counterstained in hematoxylin.

The FITC-conjugated endothelial cell marker isolectin B4 (1∶200, Vector Laboratories) and the R-PE-conjugated rat anti-mouse macrophage marker F4/80 (1∶100, Invitrogen, Carlsbad, CA) were used for double immunofluorescence staining of choroidal flat mounts (3 days after photocoagulation). The area of F4/80-positive cells within and near the laser lesions was measured using ImageJ and normalized to the size of CNV (100%).

### Quantitative Real-time Reverse Transcription Polymerase Chain Reaction (qRT-PCR)

Total RNA was extracted using TRIzol reagent (Invitrogen) from RPE–choroid complexes at various times after CNV induction. Aliquots containing 1 µg of total RNA were reverse transcribed using a first-strand cDNA synthesis kit (Roche Diagnostics GmbH, Mannheim, Germany) according to the manufacturer's instructions. qRT-PCR was performed using LightCycler (Roche Diagnostics GmbH) and SYBR green real-time PCR mix (Takara Bio, Otsu, Shiga, Japan). Target sequences were amplified using the following primer pairs: PAF-R, 5′-TTGCCTGAGCCATCCTTATT-3′ and 5′-CCTCCCACTGTGGATTGTCT-3′; VEGF, 5′-GTTCACTGTGAGCCTTGTTCAG-3′ and 5′-GTCACATCTGCAAGTACGTTCG-3′; monocyte chemotactic protein (MCP)-1, 5′-AACTCTCACTGAAGCCAGCTCT-3′ and 5′-CGTTAACTGCATCTGGCTGA-3′; IL-6, 5′-TGGAGTCACAGAAGGAGTGGCTAAG-3′ and 5′-TCTGACCACAGTGAGGAATGTCCAC-3′; and β-actin, 5′-GATGACCCAGATCATGTTTGA-3′ and 5′-GGAGAGCATAGCCCTCGTAG-3′. All estimated mRNA levels were normalized to β-actin mRNA levels.

### Enzyme-linked Immunosorbent Assay (ELISA)

RPE–choroid complexes were isolated at various time points following CNV induction and were immersed in 200 µl of tissue protein extraction reagent (T-PER; Pierce, Rockford, IL) supplemented with protease inhibitor cocktail (Halt; Pierce). The mixture was homogenized (Polytron; Kinematica AG) and clarified by centrifuging at 10,000 *g* for 5 min. VEGF, MCP-1, and IL-6 levels in the lysate were measured using the corresponding mouse VEGF, MCP-1, and IL-6 ELISA kit (R&D Systems, Minneapolis, MN) according to the manufacturer's protocols.

### Induction and Evaluation of Subretinal Fibrosis

Recently, Jo et al. successfully established a mouse model of subretinal fibrosis, resembling the fibrotic subretinal scarring observed in advanced and late-stage neovascular AMD, by introducing inflammatory macrophages into the subretinal space [26]. This model is believed to prove a significant advance in investigating molecular mechanisms for neovascular AMD and establishing new therapy besides antiangiogenic approaches. In brief, C57BL/6 mice received an intraperitoneal injection of 2.5 ml of thioglycolate, and peritoneal exudate cells (PECs) were isolated after 3 days. PECs (4×10^7^/ml) then were prepared for subretinal injection. Laser photocoagulation was performed in the posterior pole of each retina, and 0.5 µl of the prepared PECs was injected into each subretinal space using a blunt-tipped needle. Treated eyes that bled or did not exhibit focal retinal detachment were excluded. WEB2086 (1 µg) or vehicle was injected intravitreally after 2 h, and injections were repeated every other day for the duration of the study. On day 7, the mice were euthanatized, and the PEC-injected eyes were removed. After dissecting the cornea and lens from each eye, radial relaxing incisions were made in the eye cups. Staining for glial fibrillary acidic protein (GFAP) was then performed to visualize the area of residual glia on choroidal flat mounts. The GFAP area was measured to quantify subretinal fibrosis because GFAP staining detects and quantitates subretinal fibrosis effectively in this animal model [26]. Polyclonal rabbit anti-GFAP antibody (1∶400, Dako, Glostrup, Denmark) and FITC-conjugated anti-rabbit IgG (Invitrogen) were used for GFAP detection. Flat mounts were observed using a fluorescence microscope, and GFAP areas were measured using ImageJ.

### Statistical Analysis

Each result is representative of at least three independent experiments. All values represent mean ± SD. Statistical significance was assessed using Student's t-test (SPSS, Chicago, IL). *P*<0.05 was considered statistically significant.

## Results

### PAF-R Expression during Laser-induced CNV Development

**Figure 1:**
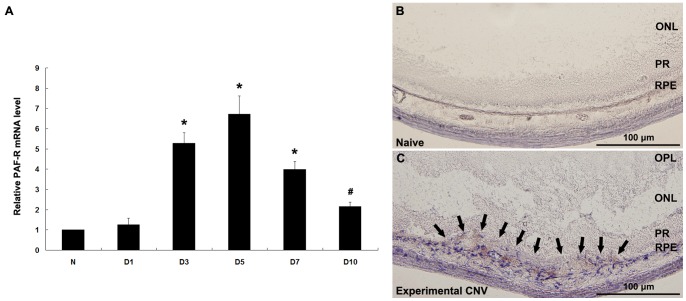
PAF-R expression during laser-induced CNV development. PAF-R mRNA expression in RPE–choroid complexes was determined by qRT-PCR analysis at various time points after photocoagulation (A). *P<0.01, ^#^ *P*<0.05 compared with the 0-hour baseline. Experiments were conducted in triplicate with similar results. Error bars indicate mean ± SD; n = 6 for each time point. Immunhistochemical analysis of PAF-R was performed in treatment-naïve eye (B) and experimental CNV tissue (arrows) on day 5 after laser treatment (C). Naïve: no laser treatment. OPL: outer plexiform layer; ONL: outer nuclear layer; PR: photoreceptors; RPE: RPE cell layer. Representative images are shown. Scale bars in B and C are 100 µm. Naïve, n = 3, experimental CNV, n = 6.

PAF-R expression during the development of laser-induced CNV was measured by qRT-PCR (Figure 1A). PAF-R mRNA level was elevated significantly (*P*<0.01) on day 3 after photocoagulation. By day 5, PAF-R mRNA peaked at levels 6.5-fold higher than the levels at the 0-h baseline (*P*<0.01). PAF-R expression decreased on day 7, but it remained elevated above the baseline on day 10 (*P*<0.05). Immunohistochemical staining confirmed the presence of PAF-R in RPE cells and choroidal endothelial cells of naïve samples (Figure 1B), and PAF-R signals became stronger in CNV regions on day 5 after photocoagulation (Figure 1C). Under high magnification, RPE-like cells and endothelial cells in CNV showed PAF-R positivity (data not shown).

### Suppression of CNV with WEB2086-induced PAF-R Blockage

**Figure 2:**
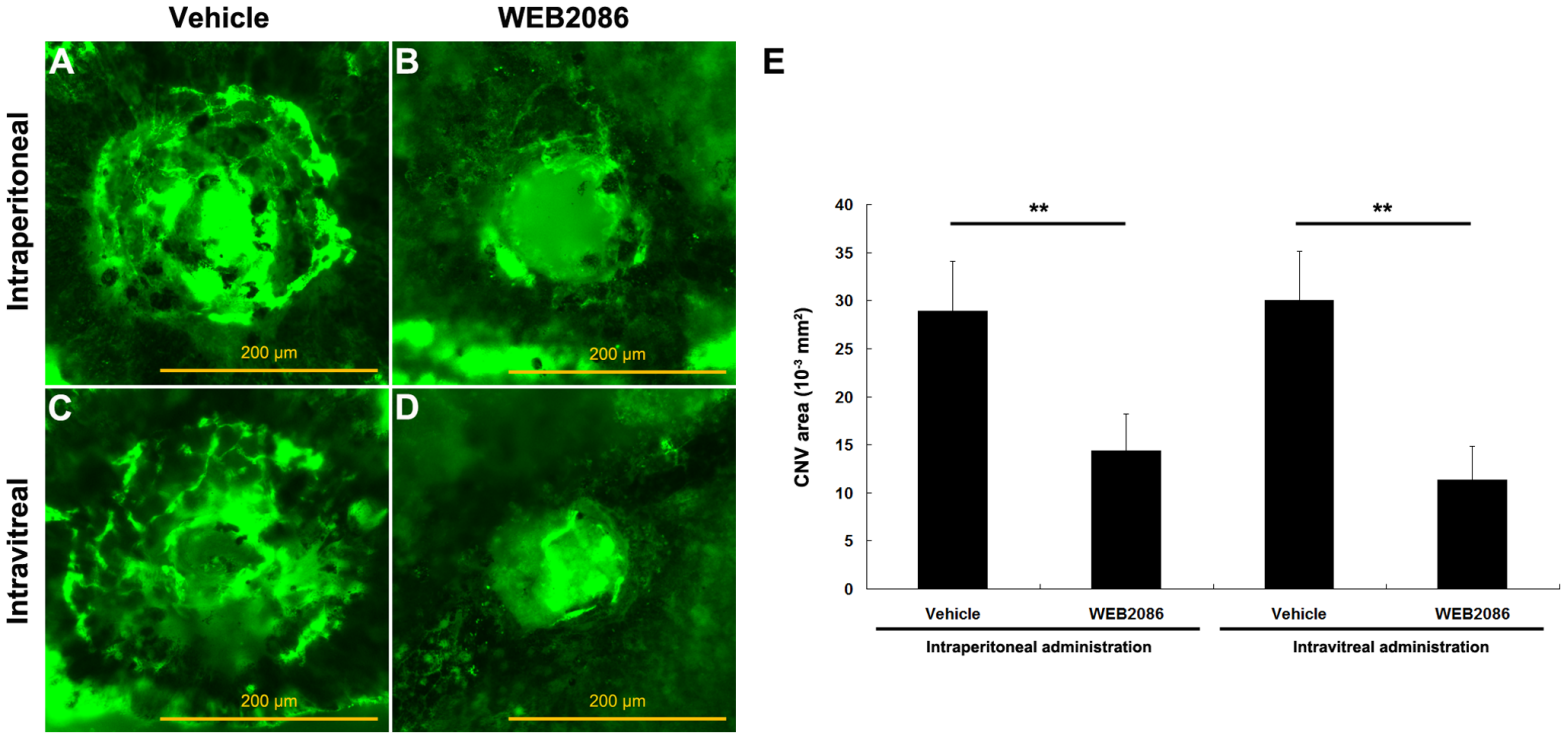
Inhibitory effects of PAF-R blockage on CNV. Representative images of fluorescein dextran perfused RPE–choroidal flat mounts of mice administered PBS vehicle only (A, C), WEB2086 intraperitoneally (B), or WEB2086 intravitreally (D) on day 10 after laser injury. The CNV area was measured quantitatively (E). **P<0.001 compared with vehicle-treated mice. Experiments were conducted in triplicate with similar results. Error bars indicate mean ± SD; intraperitoneal vehicle, n = 20, intraperitoneal WEB2086, n = 21, intravitreal vehicle, n = 20, intravitreal WEB2086, n = 22. Scale bars in A–D are 200 µm.

CNV areas on flat mounts were measured to evaluate the effects of WEB2086, a PAF-R antagonist, on CNV development. The typical features of experimental CNV in PBS-treated mice 10 days after photocoagulation included broad, flat neovascular nets (Figure 2A and 2C). In contrast, only a few new vessels were observed in mice treated with both intraperitoneal (Figure 2B) and intravitreal (Figure 2D) injections of WEB2086. Quantitative measurement suggested that both systemic and local administration of WEB2086 significantly reduced the CNV area on day 10 (*P*<0.001 for both, Figure 2E). The neovascular response in mice treated intravitreally was suppressed to a slightly greater, but non-significant, extent compared with that in mice treated intraperitoneally. Histological examination indicated no signs of retinal toxicity for intravitreal administration of WEB2086 at this dose (data not shown).

### Reduction of Macrophage Infiltration in WEB2086-treated Eyes

**Figure 3:**
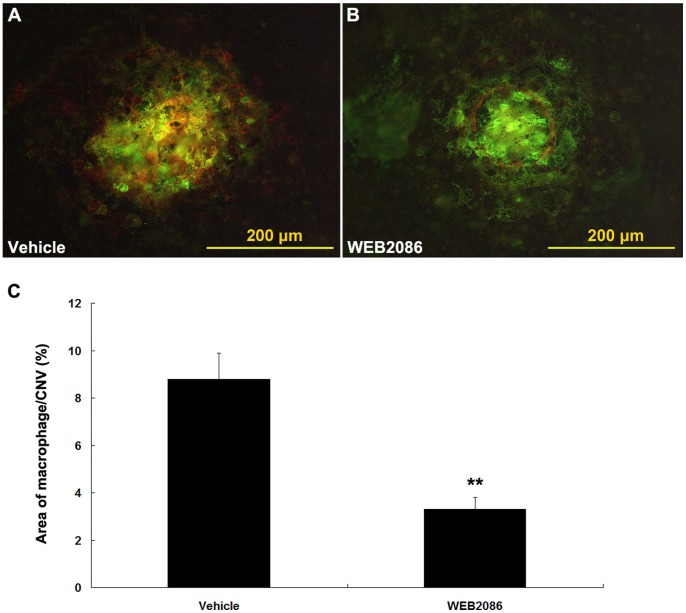
Suppressive effects of PAF-R blockage on macrophage infiltration in CNV. Immunohistochemical analysis of lesions in the choroid of vehicle- (A) or WEB2086-treated (B) mice 72 h after laser treatment. Green fluorescence from isolectin B4 indicates CNV, and red fluorescence indicates F4/80-positive macrophages. The area of F4/80-positive cells was quantified and normalized to the area of CNV (C). **P<0.001 compared with vehicle-treated mice. Error bars indicate mean ± SD; vehicle, n = 16, WEB2086, n = 16. Experiments were conducted in triplicate with similar results. Scale bars in A and B are 200 µm.

We analyzed the infiltration of macrophages into CNV lesions on choroidal flat mounts by immunostaining for F4/80, a macrophage-specific marker. F4/80-positive cells were concentrated within the laser burns and around the edges of the laser scars 72 h after laser injury (Figure 3A). No F4/80-positive cells were observed outside the laser-burned area in the choroid. In comparison, WEB2086 treatment was associated with less infiltration of F4/80-positive cells (Figure 3B). Quantitative measurement of the F4/80-positive area indicated a 57% reduction in F4/80 positivity with WEB2086 treatment (*P*<0.05, Figure 3C). These data suggest that PAF-R blockage reduces inflammation in CNV lesions.

### Inhibition of Proangiogenic and Proinflammatory Molecules with WEB2086

**Figure 4:**
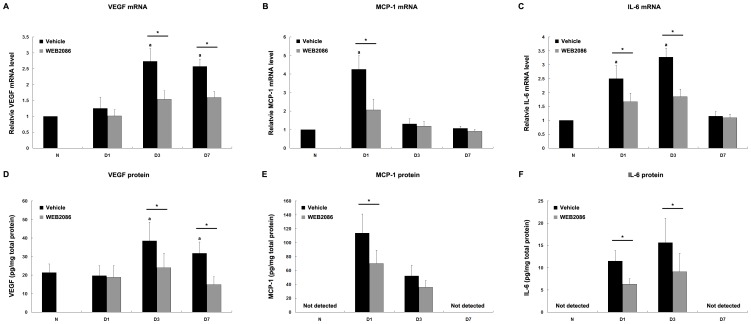
Inhibitory effects of WEB2086 on the expression of proangiogenic and proinflammatory molecules in the RPE–choroid complex. The choroidal mRNA and protein expression levels of VEGF (A, D), MCP-1 (B, E), and IL-6 (C, F) were analyzed by qRT-PCR (A–C) and ELISA (D–F) at various time points after photocoagulation. WEB2086-induced PAF-R blockage significantly suppressed the expression of these molecules, which was upregulated in the vehicle-treated mice during CNV development. *P<0.01 compared with vehicle-treated mice, ^a^ *P*<0.01 compared with the 0-hour baseline. Error bars indicate mean ± SD; vehicle, n = 8 for each molecule each time point, WEB2086, n = 8 for each molecule each time point. Experiments were conducted in triplicate with similar results.

To elucidate the molecular mechanisms involved in the regulation of CNV by WEB2086-induced PAF-R blockage, we measured the mRNA levels of proangiogenic and proinflammatory mediators in RPE–choroid complexes during CNV development. The expression levels of VEGF and the proinflammatory mediators, MCP-1 and IL-6, were significantly elevated and peaked on days 1–3, respectively, following CNV induction in the PBS-treated mice (Figure 4A–C). In the WEB2086-treated mice, the expression levels of VEGF, MCP-1, and IL-6 were significantly reduced by 44%, 52%, and 43%, respectively, at the time of peak expression for each mediator (*P*<0.01 for all). Similarly, WEB2086-induced PAF-R blockage significantly suppressed the peak protein levels of VEGF, MCP-1, and IL-6 (*P*<0.01 for all), which were upregulated after CNV induction (Figure 4D–F). These results suggest that these mediators may be targets of the PAF-R signaling pathway during CNV development.

### Suppression of Subretinal Fibrosis with WEB2086-induced PAF-R Blockage

**Figure 5:**
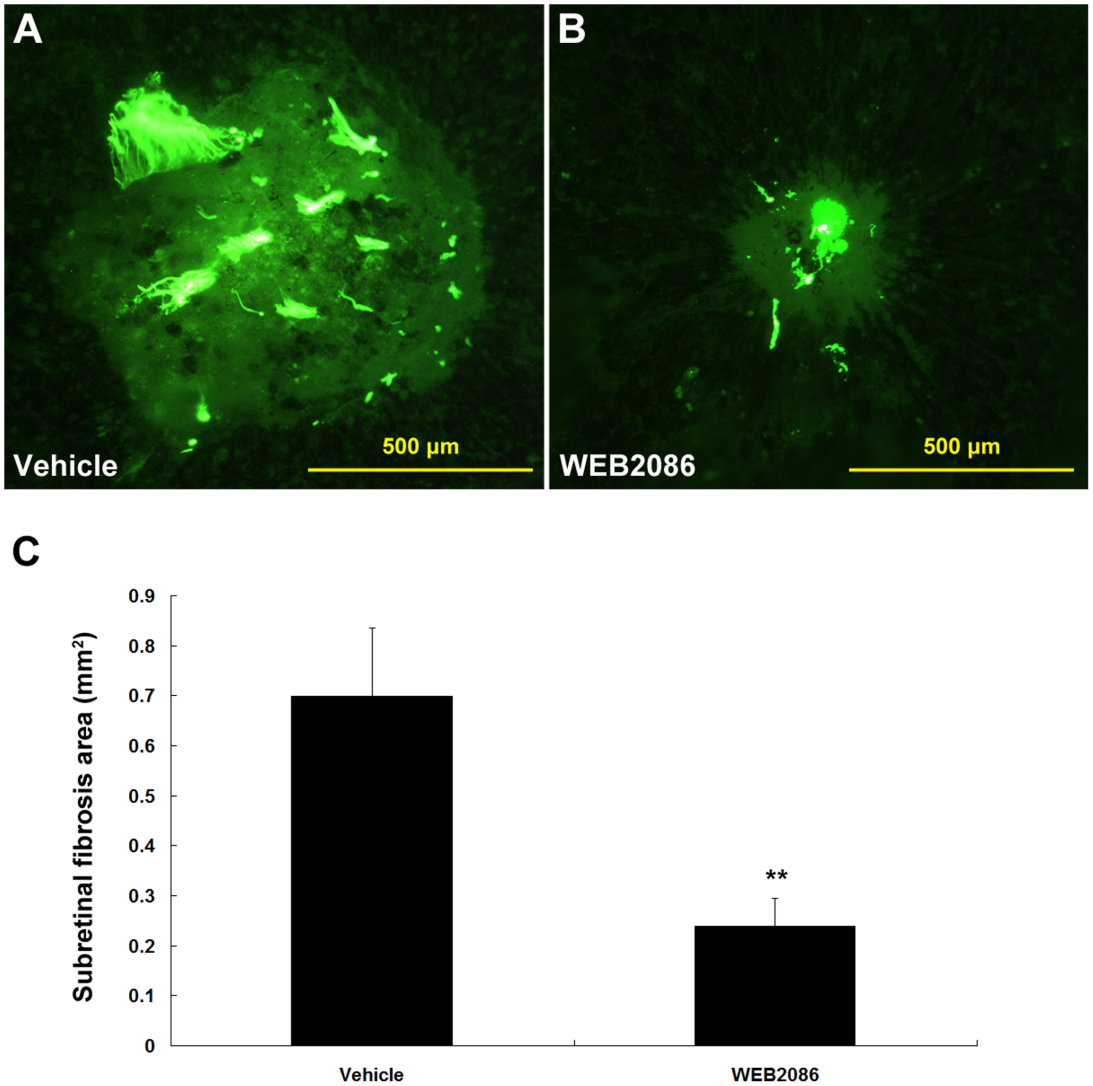
Suppression of subretinal fibrosis with WEB2086-induced PAF-R blockage in a mouse model. Representative subretinal fibrotic scarring of choroidal flat mounts from the vehicle-treated (A) or WEB2086-treated (B) mice 7 days after the induction of subretinal fibrosis. The areas of subretinal fibrosis were evaluated quantitatively by measuring the fluorescence-positive area and compared between the two groups (C). **P<0.001 compared with vehicle-treated mice. Error bars indicate mean ± SD; vehicle, n = 20, WEB2086, n = 20. Experiments were conducted in triplicate with similar results. Scale bars in A and B are 500 µm.

Recently, we successfully established a mouse model of subretinal fibrosis that exhibits fibrotic subretinal scarring similar to that observed in advanced and late-stage neovascular AMD by introducing inflammatory macrophages into the subretinal space [26]. This model facilitates investigations of the molecular mechanisms of neovascular AMD and may assist in the establishment of novel therapies that go beyond antiangiogenic approaches. Using this animal model, we evaluated whether WEB2086-induced PAF-R blockage could mitigate subretinal fibrosis. Compared with the PBS-treated mice, the WEB2086-treated mice exhibited a reduced severity of subretinal fibrosis (Figure 5A and 5B). Quantitative measurement of the fibrosis area indicated that PAF-R blockage significantly reduced subretinal fibrosis by 66% (*P*<0.001, Figure 5C). These observations suggest that PAF-R signaling may be involved in the pathogenesis of subretinal fibrosis.

## Discussion

To our knowledge, the present study is the first to characterize the relationship between PAF-R signaling and CNV. We observed PAF-R expression in laser-induced CNV and this expression increased during experimental CNV development. CNV was suppressed by blocking PAF-R using the novel receptor antagonist WEB2086. We report that the cellular and molecular mechanisms of WEB2086-induced CNV suppression included the inhibitory effects on macrophage infiltration and the expression of proangiogenic and proinflammatory molecules in the RPE–choroid complex. We also demonstrated that PAF-R blockage could suppress a model of experimental subretinal fibrosis that resembles the fibrotic subretinal scarring observed in neovascular AMD.

PAF-R has been cloned from several species and contains a seven-transmembrane domain typical of G protein-coupled receptors [16], [27]. PAF-R mRNA has been identified in various tissues, including spleen, small intestine, kidney, liver, lung, and brain, and in many cell types, including smooth muscle cells, cardiomyocytes, neutrophils, monocytes/macrophages, eosinophils, endothelial cells, and Kupffer cells [28], [29]. In the eye, PAF-R localizes to the corneal epithelium, iris/ciliary body, and retina [30]–[32] and recently was identified in RPE cells and choroidal endothelial cells [24]. Previous studies have indicated that PAF-R was upregulated during inflammatory processes, cancer progression, and wound healing process [33]–[35]. To our knowledge, the roles of PAF-R in CNV have not been addressed previously. We report here that PAF-R is upregulated during laser-induced CNV development. Laser photocoagulation is an established method for generating CNV in animal models. High laser energy causes rupture of Bruch’s membrane, and, under the influence of various angiogenic factors, an ingrowth of choroidal vessels under the RPE and into the subretinal space takes place. Although in this model, pathogenesis of the neovascularization is different from AMD, formation of CNV is believed to follow the same pattern, and identical angiogenetic factors are expressed by the RPE and endothelial cells [36]. Our observations underscore the possibility that PAF-R signaling is important in CNV.

In the present study, both systemic and local blockage of PAF-R using the novel PAF-R antagonist WEB2086 led to significant CNV suppression. Previous studies have revealed that a PAF-R blockade can inhibit endothelial cell migration and vascular permeability [19], [37]. In a corneal micropocket assay, the Matrigel model, and in an experimental tumor model, PAF-R antagonists significantly reduced VEGF-related and tumor-related angiogenesis [18], [19], [38]–[40]. WEB2086, a thieno-triazolo-diazepine, is a very potent and specific PAF-R antagonist with an affinity for PAF binding sites that is similar to the affinity of PAF itself [41]. Pharmacological data from animal studies support the possibility of using WEB2086 as a peroral, intravenous, or inhaled PAF-R antagonist at low dosage [41]. However, data are lacking regarding the pharmacological properties, toxicology, and safety profiles associated with intraocular administration of WEB2086. Our results demonstrated that intravitreal administration of WEB2086 inhibits laser-induced CNV without retinal destruction. WEB2086 easily crosses vessel walls and the mucosa of the airways [41], and we speculate that it readily penetrates the retina because of its small molecular weight (456 Da). Further study is necessary to investigate the penetration, intraocular pharmacokinetics, toxicology, and the effects on retinal ultrastructure and function with intravitreal administration of WEB2086. Our results suggest that PAF-R blockage may be a useful strategy in CNV treatment.

Macrophage accumulation in the CNV area and the expression of angiogenic cytokines, such as VEGF, are involved in CNV formation [42]–[44]. Mice subjected to pharmacological deletion of macrophages exhibited suppressed CNV and reduced VEGF, suggesting a role for macrophages as producers and regulators of angiogenic factors in CNV pathogenesis [45], [46]. In the present study, a PAF-R blockade suppressed macrophage infiltration as well as CNV development. Data of our present study are compatible with those of a recent study that observed an inhibition of macrophage infiltration and inflammatory processes following treatment with a PAF-R antagonist or in PAF-R-deficient animal in a model of renal inflammatory injury [47]. These data add to growing evidence supporting the role of chronic inflammatory processes in CNV and suggest that more research is needed to explore the therapeutic potential of anti-inflammatory therapy, such as PAF-R blockage, in AMD.

In addition to VEGF, many other angiogenic and inflammatory mediators contribute to CNV either directly via activation of their cognate receptors or indirectly via crosstalk with VEGF and other signaling pathways [48]. MCP-1, a CC chemoattractant protein, plays an important role in macrophage recruitment and is a key factor in CNV formation following laser injury. Decreased MCP-1 expression is associated with significant inhibition of macrophage infiltration and reduced CNV [49], [50]; these features also are observed in CCR2-deficient mice that lack the receptor for MCP-1 [43], [51]. IL-6 is a potent proinflammatory cytokine that may function in the pathogeneses of ocular neovascularization, such as proliferative diabetic retinopathy [52]. In experimental CNV, IL-6 expression is upregulated in the RPE–choroid complex, and antibody-based blockage of the IL-6 receptor or genetic ablation of IL-6 significantly suppress CNV [49]. We report that a PAF-R blockade significantly suppressed CNV-related molecules including VEGF, MCP-1, and IL-6. The suppression of VEGF and inflammation-related molecules associated with PAF-R blockage is consistent with the results of other studies that implicated PAF/PAF-R signaling in angiogenesis and inflammatory processes [53]–[55]. The molecular mechanisms functioning in CNV suppression by PAF-R blockage likely involve the inhibition of multiple inflammatory steps.

It is known that CNV lesions commonly evolve into scars over time as a consequence of the wound healing process. In addition, subretinal fibrosis contributes to a loss in visual acuity in neovascular AMD. A recent study documented the development or progression of submacular fibrosis following anti-VEGF therapy in patients with exudative AMD [56]. In these cases, the vascular component of AMD was treatable with anti-VEGF agents, but the visual outcome was ultimately limited by submacular fibrosis. Greater fibrotic responses after anti-VEGF therapy may result from an imbalance in the complex interactions between angiogenesis and tissue fibrosis during the wound healing process [56]. Ultimately, it is the scarring response that irreversibly damages photoreceptors; therefore, therapies that modify this response may help preserve or even rescue photoreceptors. PAF/PAF-R signaling may be a potential therapeutic target as it is believed to play a critical role in various fibrotic processes. For example, PAF signaling upregulates fibronectin expression and matrix production in tubuloepithelial cells and interstitial fibroblasts, two key cell types associated with renal fibrosis [57]. PAF-R is upregulated in experimental lung and liver fibrosis models, and PAF-R antagonists attenuate fibrotic responses [58]–[60]. These findings are consistent with our results, suggesting that WEB2086 substantially reduces subretinal fibrosis in a mouse model. As RPE cells, expressing PAF-R, have been identified not only as the central cell type in various ocular wound healing processes but also an important source of transforming growth factor-β, a key molecule, under pathologic conditions [2], [61], we hypothesize that they are involved in PAF-R blockage-induced fibrosis suppression in the animal model.

In summary, the present study is the first to demonstrate that PAF-R blockage reduces CNV and subretinal fibrosis *in vivo*. As optimal treatment modalities for neovascular AMD would target the multiple mechanisms of AMD-associated vision loss, including neovascularization, inflammation, and fibrosis, our results support PAF-R as an attractive target in the treatment of AMD, as well ad other ocular wound healing processes, such as proliferative diabetic retinopathy and retinopathy of prematurity.
